# Extra-mitochondrial citrate synthase controls cAMP-dependent pathway during sperm acrosome reaction in mice

**DOI:** 10.17912/micropub.biology.000579

**Published:** 2022-06-01

**Authors:** Woojin Kang, Daiki Katano, Natsuko Kawano, Mami Miyado, Kenji Miyado

**Affiliations:** 1 Department of Reproductive Biology, National Research Institute for Child Health and Development, 2-10-1 Okura, Setagaya, Tokyo 157-8535, Japan; 2 Department of Life Sciences, School of Agriculture, Meiji University, 1-1-1 Higashi-Mita, Kawasaki, Kanagawa 214-8571, Japan; 3 Department of Food and Nutrition, Beppu University, 82 Kita-Ishigaki, Beppu, Oita 874-8501, Japan; 4 Department of Molecular Endocrinology, National Research Institute for Child Health and Development, 2-10-1 Okura, Setagaya, Tokyo 157-8535, Japan

## Abstract

The sperm consumes adenosine triphosphate (ATP) to maintain the cellular function, viability, acrosome reaction (AR), and motility. Extra-mitochondrial citrate synthase (eCS) catalyzes citrate production in the sperm head, and thus regulates sperm function through ATP synthesis, similarly to CS. This study aimed to investigate how eCS regulates AR. Herein, acrosome-reacted (ARed) sperms were rarely detected on the zona pellucida, and spontaneous ARed sperm in *eCs* -deficient (KO) sperm remained at low levels even with induced capacitation. Retarded AR of *eCs* -KO sperm was enhanced by cyclic adenosine 3′,5′-monophosphate (cAMP) treatment. In conclusion, eCS regulates AR via a cAMP-dependent pathway, which presumably contributes to sperm metabolism.

**Figure 1. Retarded acrosome reaction (AR) in eCs -deficient (KO) sperm.**
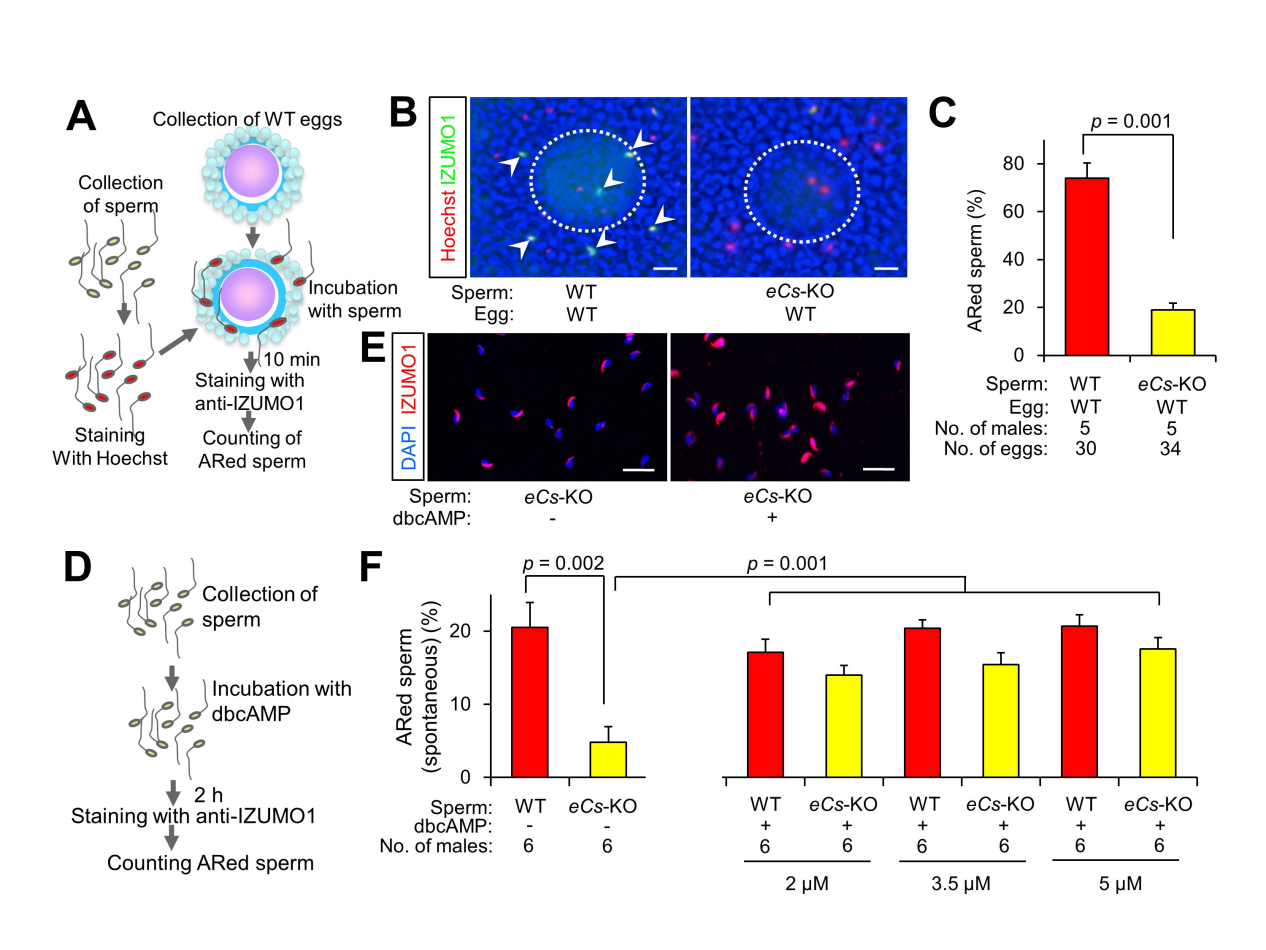
(A) Experimental flow. *eCs* -KO and wild-type (WT) sperm that were stained with Hoechst 33342 were incubated with WT eggs for 10 min and immunostained with anti-IZUMO1. (B) AR in WT and *eCs* -KO sperm. Acrosome-reacted (ARed) sperm were immunoreacted with Alexa Fluor 488-labeled anti-IZUMO1 monoclonal antibody and stained with Hoechst 33342. Arrowheads indicate ARed sperm. Dotted circles: WT eggs. Scale bar: 20 μm. (C) Percentage of ARed sperm. Values are expressed as mean ± SE. (D) Spontaneous ARed sperm. After WT and *eCs* -KO sperm were incubated without eggs in 2, 3.5, and 5 μM dibutyryl cyclic adenosine monophosphate (dbcAMP), the percentage of spontaneously ARed sperm was counted. (E) *eCs* -KO sperm incubated in 2 μM dbcAMP. Scale bar: 5 μm. (F) Percentage of spontaneously ARed sperm. Values are expressed as mean ± SE.

## Description

Sperm must undergo sequential and complex processes in the uterus and oviducts before fertilizing the eggs, including capacitation and acrosome reaction (AR) (Gervasi and Visconti 2016). Adenosine triphosphate (ATP) production through glycolysis and oxidative phosphorylation (OXPHO) is essential for maintaining sperm motility and completing AR (Stival *et al* . 2016). Citrate synthase (CS) is localized in the mitochondrial matrix, where it catalyzes the reaction between acetyl coenzyme A and oxaloacetate to form citric acid (Surpin and Chory 1997). The extra-mitochondrial form of CS (eCS) is encoded by a separate gene in mice (citrate synthase like (Csl) MGI:1919082) and expressed by alternative splicing of the *CS* gene in humans (Kang *et al* . 2020). eCS also has CS activity, implying its involvement in ATP production which is related to sperm function in the extra-mitochondrial cytoplasm (Kang *et al* . 2020). Citrate serves as an essential substrate in the tricarboxylic acid (TCA) cycle, and its subsequent complete oxidation is the major source of cellular ATP production (Iacobazzi and Infantino 2014). Consequently, the activation of TCA cycle-related enzymes that regulate cellular ATP production may be involved in controlling sperm functions (Zhu *et al* . 2019). Conceivably, eCS is involved in regulating AR in the sperm head, as citrate is an important substrate in cellular energy metabolism (Iacobazzi and Infantino 2014). However, the role of eCS in AR remains unclear.

We studied the influence of eCS deficiency on AR using IZUMO1, an integral membrane protein of the sperm that is essential for sperm-egg fusion (Inoue *et al* . 2005). Anti-IZUMO1 monoclonal antibody (mAb) can react with the sperm head only after permeabilization because IZUMO1 is present in the inner acrosomal region before AR (Inoue *et al* . 2005). However, in sperm that have undergone AR, anti-IZUMO1 mAb can react with the sperm head even without permeabilization (Inoue *et al* . 2005). As depicted in Figure 1A, Hoechst 33342-stained sperm were incubated with cumulus-intact eggs for 10 min and subjected to immunostaining with an anti-IZUMO1 mAb without permeabilization of the sperm. Although the WT sperm underwent AR successfully, it failed to undergo AR in the *eCs* -deficient ( *eCs* -KO) sperm (73.9 ± 6.4% vs. 19.0 ± 2.8%; *p* = 0.001) (Figure 1B, C).

To induce sperm AR in both humans and mice (Buffone *et al* . 2014), the elevation of intracellular calcium in the post-acrosomal region is essential and is thought to be induced by the cyclic adenosine 3′,5′-monophosphate (cAMP) signaling pathway (Breitbart 2002). To assess the involvement of the cAMP-dependent pathway in the AR of *eCs* -KO sperm, we evaluated the AR in *eCs* -KO sperm after dibutyryl cAMP (dbcAMP), a cell-permeable synthetic analog of cAMP, treatment. As depicted in Figure 1D, sperm were collected from the cauda epididymis and incubated in dbcAMP (2, 3.5, and 5 μM) for 2 h without cumulus-intact eggs. The sperm were then immunostained with anti-IZUMO1 mAb and counterstained with 4’,6-diamidino-2-phenylindole (DAPI). AR is known to occur spontaneously, even when sperm are cultured without cumulus-intact eggs (Bhakta *et al* . 2019). The percentage of sperm that underwent AR among those incubated without cumulus-intact eggs was then evaluated. Consequently, without dbcAMP treatment, the percentage of acrosome-reacted (ARed) *eCs* -KO sperm was significantly lower than that of WT sperm (4.8 ± 2.1% vs. 20.5 ± 3.4%; *p* = 0.002; Figure 1F, left). After treatment with 2 μM dbcAMP, the percentage of ARed WT sperm remained unaltered, while the percentage of ARed *eCs* -KO sperm was strikingly elevated and turned out to be comparable with that of the WT sperm (17.1 ± 1.8% vs. 14.0 ± 1.3%; Figure 1F, right). Similarly, when the sperm were treated with 3.5 and 5 μM dbcAMP, the percentage of ARed WT sperm remained unaltered, while the percentage of ARed *eCs* -KO sperm was strikingly elevated but comparable with that of the WT sperm (15.5 ± 1.6% vs. 20.4 ± 1.1%; 17.6 ± 1.6% vs. 20.7 ± 1.6%). These results indicated that cAMP signaling plays an important role in the AR of *eCs* -KO sperm.

AR retardation in *eCs* -KO sperm was linked to decreased mitochondrial activity. Mitochondrial activity has been proposed to be a useful biomarker for evaluating the fertilization ability of sperm both *in vivo* and *in vitro* (Barbagallo *et al* . 2020). In particular, the regulation of sperm motility and AR is highly dependent on mitochondrial activity (Moraes and Meyers 2018). The activities of some mitochondrial enzymes such as CS and respiratory complexes showed a high correlation with sperm motility (Ruiz-Pesini *et al* . 2000), and asthenozoospermia was the cause of the reduction in their activities. As reported earlier (Kang *et al* . 2020), mitochondrial metabolic activity is altered by reduced amounts of citrates in *eCs* -KO sperm, suggesting a possible role for eCS proteins in regulating mitochondrial activity. However, *eCs* -KO sperm exhibited normal motility, indicating no correlation between eCS and sperm motility.

It is well known that ATP is essential for sperm motility and AR (Stival *et al* . 2016). As both glycolysis and OXPHO pathways can produce ATP in sperm (du Plessis *et al* . 2015), the activities of the enzymes involved in these pathways are important for ATP production. eCS is localized in the acrosome of the sperm head, while CS is localized inside the mitochondria of the sperm midpiece. Importantly, when *eCs* -KO sperm were incubated in the presence of dbcAMP, ARed sperm levels were significantly increased, raising the possibility that cAMP plays a critical role in *eCs* -KO sperm function. The cAMP-dependent protein kinase (PKA) signaling pathway plays a central role in the regulation of energy balance and metabolism (London *et al* . 2020). Because of its location, eCS may be directly or indirectly involved in ATP-related induction of AR. It would be interesting to uncover the mechanism by which eCS regulates energy metabolism in sperm.

In conclusion, the loss of eCS resulted in retarded AR, which was rescued by dbcAMP treatment, implying that eCS regulates AR via cAMP-mediated energy metabolism. Our results contribute to the understanding of eCS-mediated energy metabolism in sperm AR induction, which is widely observed in organisms, including humans.

## Methods

**Animals**

Using the method reported in a previous study (Kang *et al* . 2020), mutant mice were generated from C57BL/6-derived embryonic stem cell clones by injecting blastocysts from C57BL/6 mice with genetically deleted *Csl* ( *eCs* ) (Csl ^tm1(KOMP)Vlcg^ ;MGI:4842999) obtained from the knockout mouse project (KOMP) repository, an NCRR-NIH-supported strain suppository. C57BL/6J mice (Japan SLC Inc., Shizuoka, Japan) were used as the controls. All the mice were housed under specific pathogen-free conditions. Food and water were provided *ad libitum* . All animal experiments were approved by the Institutional Animal Care and Use Committee of the National Research Institute for Child Health and Development (experimental number, A2004-004).

**Antibodies and reagents**

The rat anti-mouse IZUMO1 mAb required for immunostaining was kindly provided by Dr. Ikawa (Osaka University, Japan). Alexa 488- and 546-conjugated IgG were purchased from Molecular Probes (Invitrogen, Carlsbad, CA, USA) as secondary antibodies for immunohistochemistry. Nuclei were counterstained with DAPI (WAKO Pure Chemical Industries, Osaka, Japan). dbcAMP was purchased from Sigma-Aldrich (St. Louis, MO, USA).

**Sperm/cumulus penetration assay**

Cauda epididymal sperm were incubated in a 200-μL drop of TYH medium containing Hoechst 33342 (2.5 µg/mL) for 10 min at 37 °C and then capacitated by incubation in a 100-μL drop of TYH medium for 2 h at 37 °C under 5% CO _2_ . The capacitated sperm (150 sperm/µL) was mixed with the cumulus-oocyte complexes (COCs) in a 100-μL TYH drop and incubated for 10 min at 37 °C in 5 % CO _2_ . The COCs and sperm were then transferred to a 100-μL drop of fresh TYH medium and fixed by treatment with 100-µL Hanks’ balanced salt solution containing 8% paraformaldehyde and 1% polyvinylpyrrolidone (PVP) for 15 min, followed by washing with HBS containing 0.5% PVP. COCs and sperm were immunostained with Alexa Fluor 488-labeled anti-IZUMO1 mAb. The COCs were lightly squashed to accommodate them within the 80-µm space between the glass slide and the coverslip and then observed under a fluorescent microscope (KEYENCE BZ-X710, Keyence, Osaka, Japan). Fluorescent images were captured as vertical sections with 3-μm intervals and then stacked into a single picture using BZ-analyzer software (Keyence), as described previously (Kang *et al* . 2010).

**Spontaneous AR**

Cauda epididymal sperm were dispersed in a 200-µL drop of albumin-free TYH medium for 10 min and subsequently incubated in albumin-containing TYH medium at 37 °C under 5% CO _2_ . After 2 h of incubation in the absence and presence of 2, 3.5, and 5 mM dbcAMP, the sperm were transferred into a 1.5-mL microtube, washed with HBS, and fixed with 4% PFA in HBS on ice for 15 min. After fixation, the sperm were immunoreacted with an anti-IZUMO1 antibody, incubated with an Alexa Fluor 568-conjugated antibody against rabbit IgG, and counterstained with Hoechst 33342. Fluorescent images were obtained under a fluorescence microscope (KEYENCE BZ-X710).

**Statistical analysis**

Comparisons were made using one-way analysis of variance following Scheffe’s method, Mann–Whitney *U* -test, or Fisher’s exact test. Statistical significance was defined as *p* < 0.05. The results are expressed as the mean ± standard error of the mean.
